# Continuous-wave operation of 1550 nm low-threshold triple-lattice photonic-crystal surface-emitting lasers

**DOI:** 10.1038/s41377-024-01387-4

**Published:** 2024-02-05

**Authors:** Ziye Wang, Xia Liu, Pinyao Wang, Huanyu Lu, Bo Meng, Wei Zhang, Lijie Wang, Yanjing Wang, Cunzhu Tong

**Affiliations:** 1 State Key Laboratory of Luminescence and Applications, Changchun Institute of Optics, Fine Mechanics and Physics, Chinese Academy of Sciences, Changchun, 130033 China; 2 Center of Materials Science and Optoelectronics Engineering, University of Chinese Academy of Sciences, Beijing, 100049 China; 3 Central Research Institute Planning Dept, 2012 Labs, Huawei Technologies Company Ltd., Shenzhen, 518129 China

**Keywords:** Diode lasers, Semiconductor lasers, Photonic devices, Nanophotonics and plasmonics

## Abstract

Benefitting from narrow beam divergence, photonic crystal surface-emitting lasers are expected to play an essential role in the ever-growing fields of optical communication and light detection and ranging. Lasers operating with 1.55 μm wavelengths have attracted particular attention due to their minimum fiber loss and high eye-safe threshold. However, high interband absorption significantly decreases their performance at this 1.55 μm wavelength. Therefore, stronger optical feedback is needed to reduce their threshold and thus improve the output power. Toward this goal, photonic-crystal resonators with deep holes and high dielectric contrast are often used. Nevertheless, the relevant techniques for high-contrast photonic crystals inevitably complicate fabrication and reduce the final yield. In this paper, we demonstrate the first continuous-wave operation of 1.55 μm photonic-crystal surface-emitting lasers by using a ‘triple-lattice photonic-crystal resonator’, which superimposes three lattice point groups to increase the strength of in-plane optical feedback. Using this geometry, the in-plane 180° coupling can be enhanced threefold compared to the normal single-lattice structure. Detailed theoretical and experimental investigations demonstrate the much lower threshold current density of this structure compared to ‘single-lattice’ and ‘double-lattice’ photonic-crystal resonators, verifying our design principles. Our findings provide a new strategy for photonic crystal laser miniaturization, which is crucial for realizing their use in future high-speed applications.

## Introduction

Because of their extreme brightness and narrow spectral linewidth, photonic crystal surface-emitting lasers (PCSELs) have recently attracted much attention as novel semiconductor lasers^1^. These lasers use two-dimensional photonic-crystal resonators to achieve optical feedback. Light amplification and lasing are realized at the band edge by forming a broad-area standing wave in the lateral direction, with the light extracted from the normal direction by first-order diffraction. A narrow-divergence far-field pattern can be achieved due to the broad-area coherent resonance^2–4^. Recently, single-mode lasing from GaAs-based PCSELs with an emission region of 3 mm diameter was demonstrated under continuous-wave (CW) conditions^3,5^, showing an output power above 50 W and a divergence angle of 0.045°. This result indicates the enormous potential of PCSELs. In addition, the scaling property^6^ of photonic crystals makes it easy to extend the lasing wavelength^7–10^ or achieve a monolithic multiwavelength PCSEL array. In the last decade, various characteristics of PCSELs have been investigated in detail, including polarization^11–13^, beam steering^14,15^, and short pulse generation^16–18^.

The 1.55 μm PCSELs are expected to be essential for optical communications and light detection and ranging (LiDAR) due to the minimum fiber loss window and high eye-safe threshold. Nevertheless, due to their higher material absorption than GaAs counterparts, the device performance of PCSELs operating at 1.55 μm has lagged behind until now (albeit with some developments seen using 1.3 μm PCSELs^19–23^). One effective way to improve their performance is to optimize the threshold gain and output power by increasing the cavity optical feedback^2,24,25^. Therefore, photonic crystal resonators with deep holes and high dielectric contrast are usually introduced^20,21,26–28^, which inevitably increases the fabrication difficulty. In this regard, novel methods with flexible and accessible optical feedback control are needed.

Here, we propose a triple-lattice photonic crystal resonator to enhance the in-plane optical feedback by appropriately designing the unit cell^29^. The proposed structure is regarded as a nest of three lattice point groups with modulated mutual interactions. The 180° coupling was enhanced threefold when the nested lattices are arranged for constructive interaction. Therefore, the device realizes more robust optical feedback at a lower threshold, distinct from previous single-lattice and double-lattice photonic-crystal resonators. In this paper, we first present the concept of a triple-lattice photonic-crystal resonator and analyze its underlying principles. Next, the device structure is described in detail. Finally, we discuss the device characteristics and calculate the optical loss of various photonic crystal resonators for comparison by three-dimensional coupled-wave theory (3D-CWT)^30^.

**Fig. 1:**
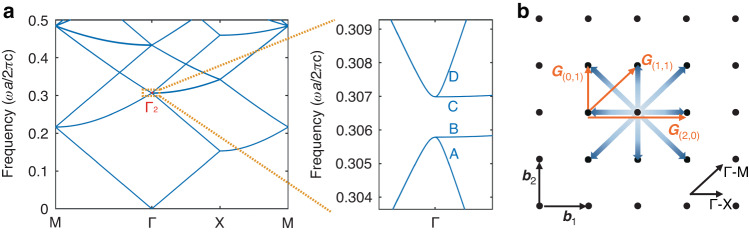
Operating principles of the photonic-crystal resonator. **a** (Left) Band structure for a square-lattice photonic crystal with TE polarization. (Right) Magnified band structure near the Γ_2_-point. **b** Bloch wave state represented by wave vectors (blue arrows) in reciprocal space. The orange arrows indicate reciprocal lattice vectors corresponding to the coupling among wave vectors

The operating principles of the investigated PCSELs are illustrated in Fig. 1. Similar to edge-emitting lasers, PCSELs realize vertical optical confinement by a multilayer epitaxial structure (see Supplementary Table S1), which supports only a single fundamental guided mode. To obtain the strongest in-plane optical feedback, a photonic crystal is embedded inside the waveguide layer adjacent to multi-quantum wells (MQWs). This layer is denoted the photonic crystal layer. We adopt a square-lattice photonic crystal. Figure 1a shows the corresponding photonic band structure calculated by the plane-wave expansion method (PWEM)^31,32^. The red point assigned as Γ_2_ is the singularity point where two-dimensional resonance and vertical lasing occur. At the Γ_2_ point, four band-edge modes are individually referred to as modes A, B, C, and D, according to their field distribution. The following theoretical and experimental analyses are all focused at the Γ_2_ point. Figure 1b shows the Bloch wave state represented by wave vectors in the reciprocal space of the square-lattice photonic crystal. It intuitively illustrates the in-plane coupling relationship of fundamental waves propagating inside the PCSELs. The reciprocal lattice vectors are given by

1 $${{\bf{G}}}_{(m,n)}=\left(m\frac{2\pi }{a},n\frac{2\pi }{a}\right)$$

where, *m* and *n* are integers. ***b***_1_ and ***b***_2_ are the primitive translation vectors of the reciprocal lattice. The blue arrows represent primary propagating waves in the resonator, with four fundamental waves propagating along the Γ-X direction and four higher-order waves propagating along the Γ-M direction. They couple with each other via reciprocal lattice vectors^33^. Here, we focus on transverse-electric-like (TE-like) modes because of the compressively strained quantum wells with TE gain. Among these couplings, the 180° couplings between the four fundamental waves dominate the optical feedback strength and thus the in-plane optical loss. They are directly achieved by reciprocal lattice vectors ${{\boldsymbol{G}}}_{(m,n)}|_{\sqrt{{m}^{2}+{n}^{2}}=2}$. Moreover, the orthogonal (or 90°) couplings among fundamental waves also contribute to the 2D resonance. Although they seem to be achieved directly by ***G***(±1,±1) and ***G***(±1,∓1), these direct couplings are essentially forbidden because the polarizations of two orthogonal waves are perpendicular to each other. They can only be indirectly achieved by reciprocal lattice vectors, such as ***G***_(0,±1)_ and ***G***_(±1,0)_, through high-order waves. Even so, all these couplings are considered in the calculation.

## Results

### Triple-lattice photonic-crystal resonator

**Fig. 2:**
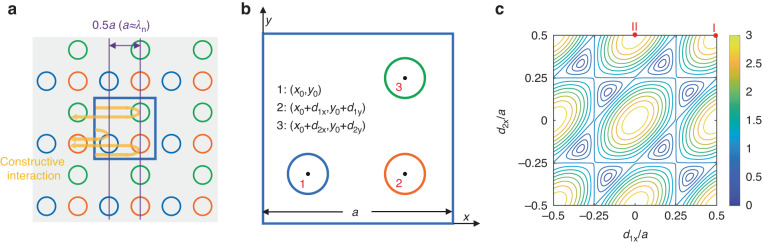
Design of the triple-lattice photonic-crystal resonator. **a** Schematic of the triple-lattice photonic-crystal resonator, composed of three lattice point groups (indicated in different colors). Yellow arrows indicate the propagating waves diffracted by the lattice. The blue square shows a unit cell. The lattice constant *a* is approximately equal to the wavelength in the material, *λ*_n_. **b** Unit cell of the triple-lattice photonic crystal. The centers of the holes are marked as points 1–3, the coordinates of which are given accordingly. **c** |*A*_2,0_| as a function of *d*_1x_ and *d*_2x_. Two of the maximum points are marked as points I and II

To enhance the strength of 180° coupling, we proposed a triple-lattice photonic-crystal resonator, as shown in Fig. 2a. The triple-lattice photonic-crystal resonator is regarded as a nest of three single-lattice point groups with the same lattice constant *a*. In other words, each unit cell of the triple-lattice photonic crystal has three holes, which are separated by half wavelengths in the *x* and *y* directions. Therefore, the optical path difference between the propagating waves diffracted by these nested lattices is an integral multiple of the wavelength, which causes enhanced optical feedback. Figure 2b shows the details of the unit cell. The centers of the three holes are marked as points 1 to 3, with corresponding coordinates (*x*_0_,*y*_0_), (*x*_0_ + *d*_1x_,*y*_0_ + *d*_1y_), and (*x*_0_ + *d*_2x_,*y*_0_ + *d*_2y_), respectively.

The coupling of propagating waves is determined by the dielectric function of photonic crystals. We first consider a single lattice and define the change in the dielectric constant relative to the high-dielectric background as *ε*(*x*,*y*). This lattice is expressed using a Fourier expansion as:

2 $$\varepsilon (x,y)=\mathop{\sum }\limits_{m,n}{F}_{m,n}\exp\left (j\frac{2\pi m}{a}x+j\frac{2\pi n}{a}y\right)$$

where *m* and *n* are integers and *F*_*m*,*n*_ are Fourier coefficients. According to coupled-wave theory, the coupling strength is proportional to the Fourier coefficient amplitude. Following the case of a single lattice, for the triple-lattice photonic crystal, the dielectric function is expressed as:

3 $$\begin{array}{l}\varepsilon (x,y)+\varepsilon (x-{d}_{1x},y-{d}_{1y})+\varepsilon (x-{d}_{2x},y-{d}_{2y})\\\qquad\qquad =\,\mathop{\sum }\limits_{m,n}\left\{1+\exp \left(-j\frac{2\pi m}{a}{d}_{1x}-j\frac{2\pi n}{a}{d}_{1y}\right)\right.\\\left.\qquad\qquad+\,\exp \left(-j\frac{2\pi m}{a}{d}_{2x}-j\frac{2\pi n}{a}{d}_{2y}\right)\right\}{F}_{m,n}\exp \left(j\frac{2\pi m}{a}x+j\frac{2\pi n}{a}y\right)\\ \qquad\qquad=\,\mathop{\sum }\limits_{m,n}{A}_{m,n}\cdot {F}_{m,n}\exp \left(j\frac{2\pi m}{a}x+j\frac{2\pi n}{a}y\right)\end{array}$$

where the three terms on the left side of the equation represent the dielectric functions of the three nested lattices. *A*_*m*_,_*n*_ represents the modulation of the Fourier coefficients compared to the single-lattice structure, with the value depending on the order of Fourier expansion and the separation of nested lattices. For the 180° coupling of the fundamental waves, we focus on the terms with${A}_{m,n}|_{\sqrt{{m}^{2}+{n}^{2}}=2}$. Specifically, we obtain the corresponding modulation factor:

4 $${A}_{2,0}=1+\exp\left (-j\frac{4\pi }{a}{d}_{1x}\right)+\exp\left (-j\frac{4\pi }{a}{d}_{2x}\right)$$

Figure 2c illustrates the relationship between the amplitude of the modulation factor |*A*_2,0_| and the separation of nested lattices in the *x* direction. The data show that |*A*_2,0_| can be adjusted from 0 to 3 by changing *d*_1x_ and *d*_2x_. When |*A*_2,0_| is 3, the strongest 180° coupling via ***G***_(2,0)_ could be expected. To obtain the maximum optical feedback and lowest lasing threshold, the position of the holes should be arranged according to these maxima. For instance, point I in Fig. 2c indicates that the *x* coordinates of the second and third holes are both *x*_0_ + *a*/2, while point II indicates that these x-coordinates are *x*_0_ and *x*_0_ + *a*/2, respectively. Because the square lattice has C_4_ symmetry, rules derived for the coupling via ***G***_(2,0)_ are also suitable for those coupling via ***G***_(−2,0)_, ***G***_(0,2)_, and ***G***_(0,−2)_. Based on this analysis, we set the value of (*d*_1x_,*d*_2x_) as (*a*/2,*a*/2) and the value of (*d*_1y_,*d*_2y_) as (0,*a*/2) to obtain the strongest in-plane coupling. The corresponding coordinates of the air holes are shown in Fig. 2b. A theoretical comparison between the triple lattice and other photonic crystal resonators is shown in Supplementary Fig. S1. Note that, only the 180° couplings are considered significant to determine the optical feedback in our design. This can be validated by the huge difference between the coupling coefficients of various process in our triple-lattice photonic-crystal resonator. For instance, the maximal coupling coefficient of 180° couplings between fundamental waves is about 253 cm^−1^, while that of the high-order couplings is only 9.6 cm^−1^, as explained in Supplementary Fig. S2.

### Structure of the triple-lattice PCSEL

**Fig. 3:**
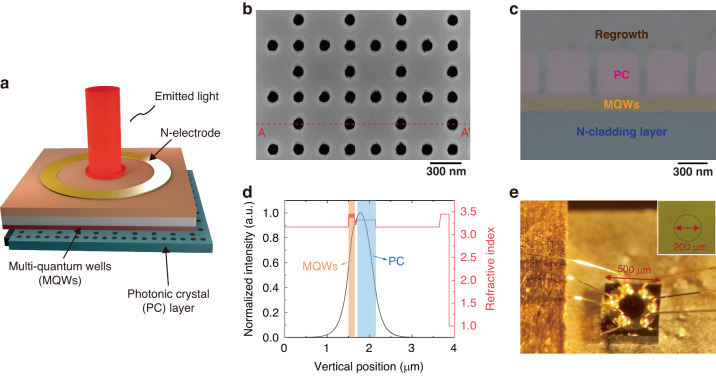
Structure of the triple-lattice photonic-crystal surface-emitting laser (PCSEL). **a** Schematic of the PCSEL structure. **b** Top-view scanning electron microscope image of the triple-lattice photonic-crystal resonator. **c** Cross-sectional SEM image of the resonator along AA’ in **b** after epitaxial regrowth. **d** The refractive index profile with the corresponding optical field distribution along the crystal growth direction. **e** Images of the laser chip bounded to a thermally conductive submount with p-side down. The inset shows the p-side of the laser chip

The structure of PCSELs is schematically illustrated in Fig. 3a. We adopt all-semiconductor photonic-crystal resonators in this work, which have better manufacturability and reliability than void-containing resonators^34^. Details of the epitaxial structure are given in Supplementary Table S1. Figure 3b shows the top-view scanning electron microscope (SEM) image of the triple-lattice photonic-crystal resonator, of which the cross-sectional image after regrowth is given in Fig. 3c. The holes are filled by low-dielectric InP, with a hole diameter of 90 nm, a height of 430 nm, and a lattice constant of 474 nm. This indicates that the filling factor of the photonic crystal is approximately 8.5%, which is defined by the area ratio of holes to the whole unit cell. Figure 3d shows the optical field distribution of the fundamental transverse mode and the refractive index profile along the crystal growth direction. The average refractive index of the photonic crystal layer used in the calculation is defined as:

5 $${n}_{{\rm{ave}}}=\sqrt{FF\ast {{n}_{{\rm{h}}}}^{2}+(1-FF)\ast {{n}_{{\rm{b}}}}^{2}}$$

where *n*_h_ and *n*_b_ are the refractive index of holes and background, respectively, and *FF* is the filling factor^35^. The thickness of the photonic crystal layer is set as 430 nm, corresponding to the SEM image after regrowth. The optical confinement factors in the photonic crystal layer and MQWs are 52 and 6%, respectively. Figure 3e is a picture of the fabricated device, with a mesa length of 500 μm. The right-upper inset shows the p-mesa with a diameter of 220 μm, while the contact window has a diameter of 200 μm. The n-electrode includes a ring window with an inner diameter of 240 μm that allows for transmission of emitted light. The substrate was thinned to 180 μm, with a free carrier absorption of approximately 10.2%.

### Characterization of the triple-lattice PCSEL

**Fig. 4:**
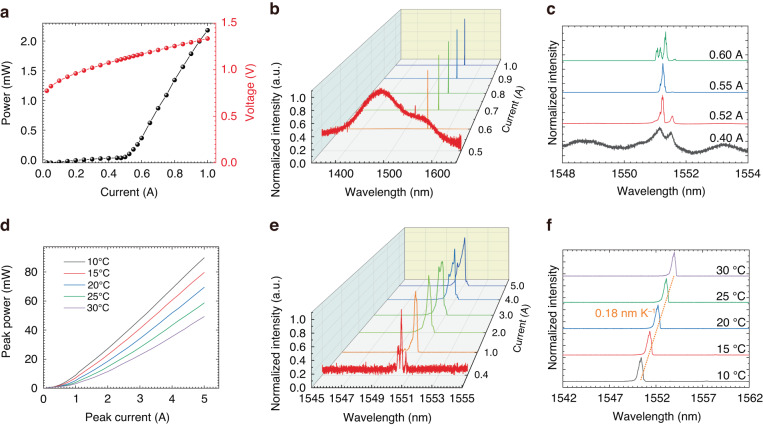
Lasing characteristics of the triple-lattice PCSEL. **a** Light-current-voltage characteristics under continuous-wave (CW) conditions, where the temperature was maintained at 10 °C during measurements. **b** Emission spectra at various injection currents under CW conditions. **c** Magnified spectra near the peak wavelength around the threshold. **d** Light-current characteristics at various temperatures under pulsed conditions. **e** Emission spectra of pulsed lasers at various injection currents at 10 °C. **f** Temperature dependence of the emission spectra at an injection current of 1 A

The characteristics of the fabricated triple-lattice PCSEL are shown in Fig. 4. Figure 4a gives the light-current-voltage characteristics of the device under CW conditions. An output power of 2.1 mW is obtained at 1 A, with a threshold current of 0.52 A (1.66 kA cm^−2^ current density). The differential resistance of the device is approximately 0.4 Ω. The measurement details are shown in Supplementary Fig. S3. The emission spectra at various injection currents are shown in Fig. 4b. To check whether the device only works at the Γ_2_ point, spectra with a wide range (1350–1650 nm) were recorded. No other resonant peaks except at approximately 1551 nm were observed, in agreement with the calculation. To characterize the lasing behavior in detail, zoomed-in spectra around the threshold are shown in Fig. 4c, which shows that multimode lasing operation occurs at a current of 0.60 A. Figure 4d–f shows the device performance under pulsed conditions. The repetition frequency and pulse width are 1 kHz and 200 ns, respectively. The PCSEL chip shows a maximum peak power of 89 mW at 5 A, as shown in Fig. 4d. The triple-lattice PCSELs work in multi-mode with a side-mode suppression-ratio (SMSR) of 3.2 dB at injected current of 5 A, as shown in Fig. 4e. This could be attributed to the smaller threshold gain difference between the fundamental and other modes when enhancing the 180° coupling^2,3^. The slope efficiency decreases from 0.02 W A^−1^ to 0.013 W A^−1^ with increasing temperature, with a wavelength tuning coefficient of 0.18 nm K^−1^, as shown in Fig. 4f. In Supplementary Table S2, a comparison between our work and those in the literature is presented. Additionally, some calculation results for void-containing PCSELs are provided in Supplementary Fig. S4. We believe that PCSELs with smaller cavity and lower threshold current can be obtained by combining the advantages of strong feedback of triple-lattice photonic crystal and high dielectric contrast of void-containing resonator, if the manufacturability and reliability are solved in future.

**Fig. 5:**
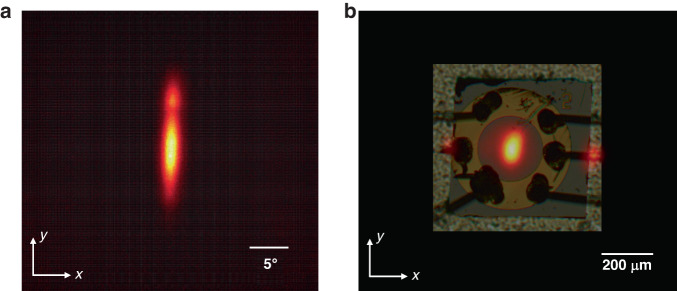
Lasing emission patterns of the triple-lattice PCSEL. **a** Far-field and **b** near-field patterns of the fabricated PCSEL

The far-field pattern of the PCSEL under CW operation is shown in Fig. 5a. The divergence angles in the *x* and *y* directions are 3° and 17°, respectively, as estimated from the 1/e^2^ width. More results at various injection currents are shown in Supplementary Fig. S5. The elliptical beam pattern is attributed to the multi-mode lasing and nonuniform near-field distribution. The optical microscope image of the PCSEL is overlapped on the near-field pattern for ease of analysis, as shown in Fig. 5b. A vertical laser beam is emitted from the central light-output window as expected. However, part of the light leaking from the edges of the device is also observed in the near-field pattern. This result indicates that some optical feedback occurs at the cleaved facet in this direction, thus affecting the field distribution intrinsic to the photonic-crystal resonator, as indicated in Supplementary Fig. S6.

**Fig. 6:**
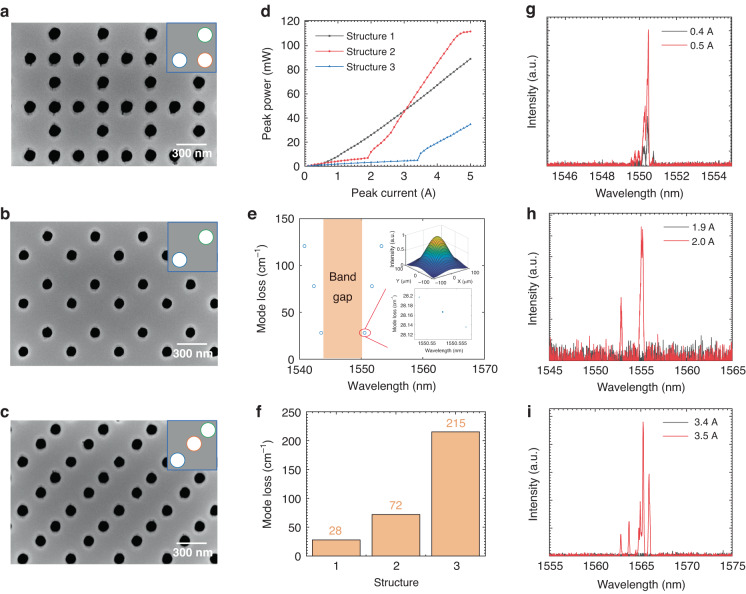
Characteristics of PCSELs with different photonic crystal resonators. **a**–**c** Top-view SEM images of the photonic crystal resonators (assigned as structures 1–3, respectively). The right-upper insets are schematics of their unit cells. **d** Light-current characteristics under pulsed conditions. **e** The calculated optical loss of the eigenmodes as a function of wavelength for structure 1. Right-down inset: the magnified image. Right-up inset: the field intensity envelope of the lowest-threshold mode. **f** Comparison of optical loss of the lowest-threshold modes. **g**–**i** Emission spectra around the threshold of structures 1–3, respectively, under pulsed conditions

To demonstrate the enhanced feedback of our design, PCSELs with three different photonic crystal resonators (assigned as structures 1 to 3) are shown in Fig. 6. Figure 6a shows the aforementioned triple-lattice photonic-crystal resonator. The nested lattices are arranged to increase the in-plane feedback by mutually constructive interaction. The coordinates of the three holes in the unit cell are (−0.25*a*, −0.25*a*), (0.25*a*, −0.25*a*), and (0.25*a*, 0.25*a*), assuming the origin at the center of the unit cell. Fig. 6b shows a double-lattice photonic-crystal resonator that aims to enhance the in-plane feedback. The coordinates of the two holes in the unit cell are (−0.25*a*, −0.25*a*) and (0.25*a*, 0.15*a*). The second hole intentionally deviates from the most symmetric position, avoiding forming a centered-rectangular lattice. Figure 6c shows a triple-lattice photonic-crystal resonator designed for reduced in-plane optical feedback by mutually destructive interaction. The coordinates of the three holes in the unit cell are (−0.37*a*, −0.37*a*), (0,0), and (0.3*a*, 0.3*a*), corresponding to the minima in Fig. 2c. As shown in Fig. 6d, the threshold currents of the three PCSELs are 0.52 A, 2.0 A, and 3.5 A, respectively. PCSEL consisting of structure 1 has the lowest threshold, as expected. In Fig. 6e, we calculate the eigenmodes of structure 1 by 3D-CWT. In the calculation, the sidewall of the holes is assumed to be vertical for simplification. The optical loss of the lowest-threshold mode is approximately 28 cm^−1^, while those of the other two structures are 72 cm^−1^ and 215 cm^−1^, respectively, as shown in Fig. 6f. The calculated and experimental results are in good agreement. The spectra of these PCSELs around the threshold are also shown in Fig. 6g–i. Notably, some imperfections on the sidewall of the photonic crystal were introduced because of the large-current exposure and dry etching process in the fabrication. Although they bring about little changes to the total optical loss, they will have side effects on the stable operation of the lasing mode, as explained in Supplementary Fig. S7.

## Discussion

Here, we have proposed a triple-lattice photonic-crystal resonator to considerably enhance the in-plane optical feedback of PCSELs. By appropriately designing the mutual interaction of the nested lattices, the 180° coupling has been increased by threefold with respect to that of the single-lattice case. The data show that the enhancement of the in-plane optical feedback significantly reduces the lasing threshold compared with the double lattice PCSELs. Devices with a lasing wavelength of 1.55 μm have shown a peak power of 89 mW and a CW output power of 2.1 mW at 10 °C. Notably, the proposed structures are also suitable for void-containing PCSELs with higher dielectric contrast. Our results present an opportunity for InP-based high-speed 1.55 μm PCSELs, which are expected to play an essential role in high-speed optical communication and LiDAR applications^36^.

## Materials and methods

### Fabrication

The device was fabricated using a metal-organic chemical vapor deposition (MOCVD) regrowth technique. First, an n-InP cladding layer, an active layer containing InAlGaAs MQWs, a p-InAlAs electron blocking layer and a p-InAlGaAs layer were grown on the n-InP substrate. The photonic crystal structure was then fabricated in a square region with a side length of 300 μm on top of the p-InAlGaAs layer by electron beam lithography and inductively coupled plasma etching. The etching depth of the holes is 375 nm. Next, 50 nm p-InAlGaAs layer and 30 nm grading layer were grown on the photonic crystal layer by MOCVD, followed by a p-InP cladding layer and a p-InGaAs contact layer. After regrowth, we fabricated a circular mesa by wet etching using H_2_O_2_/HCl/HBr solutions. Then, a 300 nm SiO_2_ electrical insulating layer was deposited, and a circular contact window with a diameter of 200 μm was opened by reactive ion etching. Finally, Ti-Pt-Au and Ni-Au/Ge-Ni-Au were deposited as the p-electrode and n-electrode, respectively. The ring window was formed by a lift-off process. More details of the fabrication process are illustrated in Supplementary Fig. S8.

### Measurement

The chips are soldered on Cu submounts with p-side down. The temperature was controlled by thermoelectric cooling (TEC) during measurements. The light-current characteristics were measured by an integrating sphere photodiode power sensor (Thorlabs S148C), which was placed in front of the ring window normal to the surface of the PCSEL. The emission spectra were recorded by an optical spectrum analyser (Yokogawa AQ6370C) with a resolution of 0.02 nm. The far-field and near-field patterns are captured by a CCD camera (Spiricon SP503U-1550).

### Supplementary information

Supplementary Information for Continuous–wave operation of 1550 nm low-threshold triple-lattice photonic-crystal surface-emitting lasers
