# Supplementary material for: Continuous-wave operation of 1550 nm low-threshold triple-lattice photonic-crystal surface-emitting lasers
Source: Light Sci Appl. 2024 Feb 5;13:44. doi: 10.1038/s41377-024-01387-4 (PMC11251162; doi:10.1038/s41377-024-01387-4)
Supplement: Supplementary file 1 — Supplementary Information for Continuous–wave operation of 1550 nm low-threshold triple-lattice photonic-crystal surface-emitting lasers [file 41377_2024_1387_MOESM1_ESM.docx]

**Supplementary Information for**

**“Continuous–wave operation of 1550 nm low-threshold triple-lattice photonic-crystal surface-emitting lasers”**

**Ziye Wang^1,2^,** **Xia Liu^3^, Pinyao Wang^1,2^, Huanyu Lu^1^, Bo Meng^1^, Wei Zhang ^1,2^, Lijie Wang^1^,
Yanjing Wang^1^ and Cunzhu Tong^1,*^**

*1* *State Key Laboratory of Luminescence and Applications, Changchun Institute of Optics, Fine Mechanics and Physics, Chinese Academy of Sciences, Changchun 130033, China*

*2* *Center of Materials Science and Optoelectronics Engineering, University of Chinese Academy of Sciences, Beijing 100049, China*

*3 Central Research Institute Planning, 2012 Labs, Huawei Technologies Company Ltd., Shenzhen,518129, China.*

********Corresponding author:* [*tongcz@ciomp.ac.cn*](mailto:tongcz@ciomp.ac.cn)

**Table S1 Epitaxial structure of the 1.55 μm PCSEL.**

| **Layer** | **Function** | **Composition** | **Thickness (nm)** | | **Doping level**  **(cm^-3^)** | **Doping type** |
| --- | --- | --- | --- | --- | --- | --- |
| 10 | p-contact layer | In_0.53_Ga_0.47_As | 200 | | 1×10^19^ | P |
| 9 | grading layer | InP→  In_0.53_Ga_0.47_As | 30 | | 1×10^18^ | P |
| 8 | p-cladding layer | InP | 300 | | 1×10^18^ | P |
|  |  |  | 300 | | 8×10^17^ |  |
|  |  |  | 300 | | 6×10^17^ |  |
|  |  |  | 300 | | 4×10^17^ |  |
|  |  |  | 300 | | 2×10^17^ |  |
| 7 | grading layer | In_0.52_Al_0.24_Ga_0.24_As  →InP | 30 | | 2×10^17^ | P |
| 6 | regrowth | In_0.52_Al_0.24_Ga_0.24_As | 50 | | 2×10^17^ | P |
| 5 | photonic crystal layer | In_0.52_Al_0.24_Ga_0.24_As | 350 | | 2×10^17^ | P |
| 4 | grading layer | In_0.52_Al_0.48_As→  In_0.52_Al_0.24_Ga_0.24_As | 30 | | 2×10^17^ | P |
| 3 | electron blocking layer | In_0.52_Al_0.48_As | 30 | | 2×10^17^ | P |
| 2 | multi-quantum wells (MQWs) | In_0.44_Al_0.13_Ga_0.43_As | 10 | ×9 |  | undoped |
|  |  | In_0.68_Al_0.05_Ga_0.27_As | 6 |  |  |  |
|  |  | In_0.44_Al_0.13_Ga_0.43_As | 10 | |  |  |
| 1 | n-cladding layer | InP | 1500 | | 2×10^18^ | N |
| 0 | substrate | InP |  | |  | N+ |

**
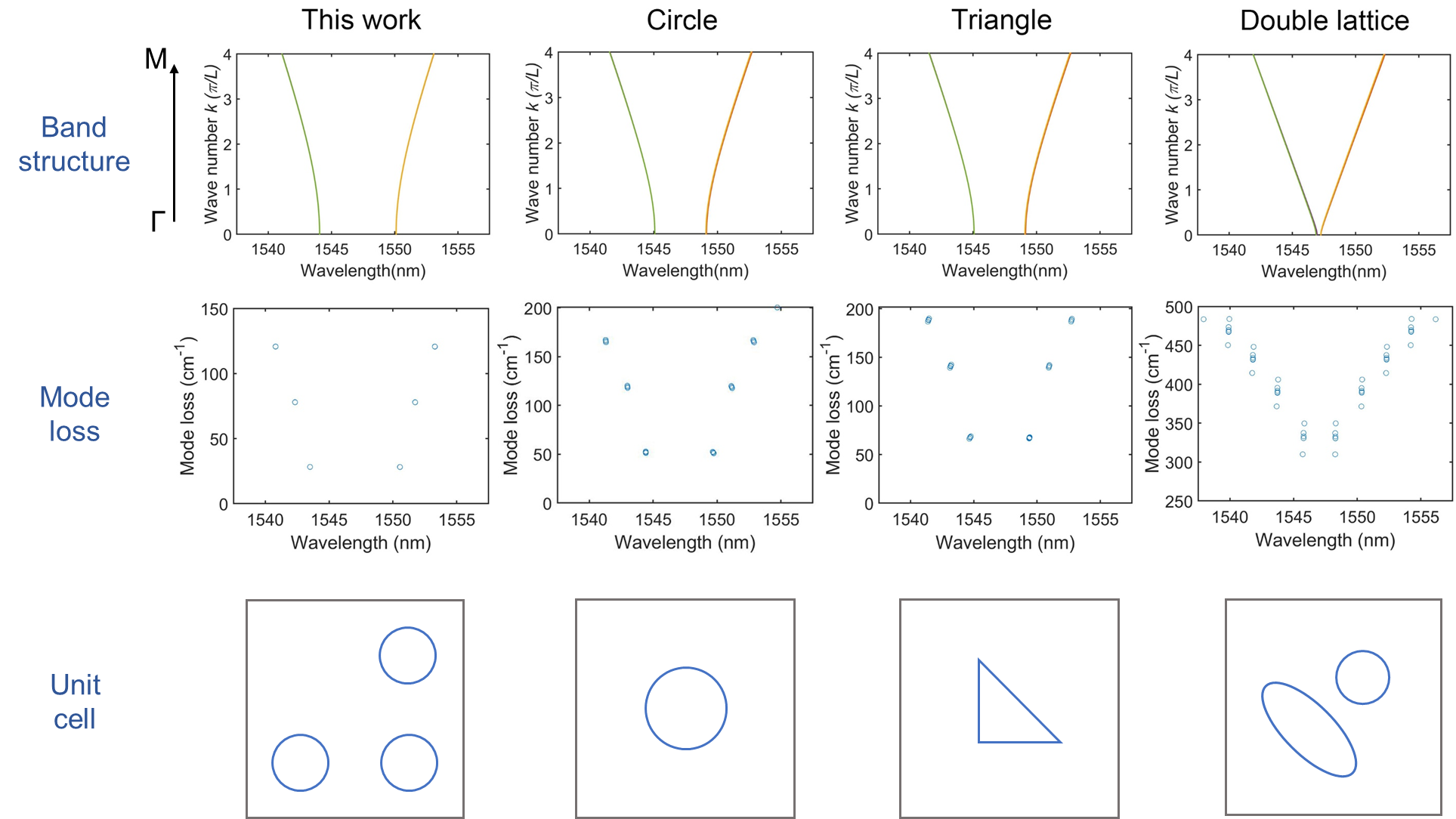
**

**Fig. S1 A theoretical comparison between our work and other photonic-crystal resonators.**

The characteristics of all-semiconductor PCSELs^1^ with different unit cells are compared theoretically. The filling factors (area ratio of the holes to the whole unit cell) of all these structures are set as 8.5%. The vertical field distribution is the same as Fig. 3d in the main text. Photonic band structures of the PCSELs are calculated by plane-wave expansion method (PWEM), and only the bands in the vicinity of second-order Γ point are shown. The range of the wave number is set as 0-4π/*L* along the Γ-M direction. The photonic-crystal resonator is regarded as a square region in the calculation for simplification, and *L* is its side length. The value of L is 200 μm, corresponding to 422 periods. The total optical loss of eigenmodes is calculated as a function of wavelength by three-dimensional coupled-wave theory (3D-CWT). The mode loss is the sum of in-plane optical loss and vertical-radiation loss. However, the in-plane optical loss is the dominant factor in these resonators. The fundamental and high-order modes correspond to the points in the band structure with wave number of *m*π/*L* (m=1, 2, 3, …)^2^. Among these four resonators, the triple-lattice photonic-crystal resonator adopted in our work enables PCSELs to have the lowest threshold compared to the others, as expected. This is due to its strongest in-plane optical feedback, hinted by the wider photonic band gap^3,4^.


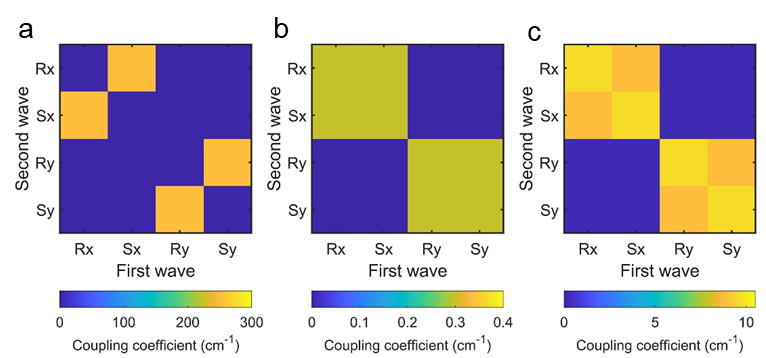


**Fig. S2 The coupling coefficient between the fundamental waves.** **a** 180° direct couplings *κ*_1D_. **b** Indirect coupling via radiative waves *κ*_rad_. **c** Indirect coupling via high-order waves *κ*_2D_.

According to the coupled-wave theory, the resonant field of a two-dimensional photonic crystal is mainly composed of four fundamental waves, namely, Rx, Ry, Sx, and Sy, whose amplitudes reflect the intensity of the standing-wave field. The 180° coupling refers to the direct couplings between Rx and Sx, or Ry and Sy, which are similar to the coupling between reverse transmission waves in the analysis of grating. We denote its strength as *κ*_1D_. However, some other indirect couplings are also contributing to forming the 2D resonance. Specifically, we denote them as *κ*_rad_ and *κ*_2D_ according to their relationship with radiative or high-order waves.


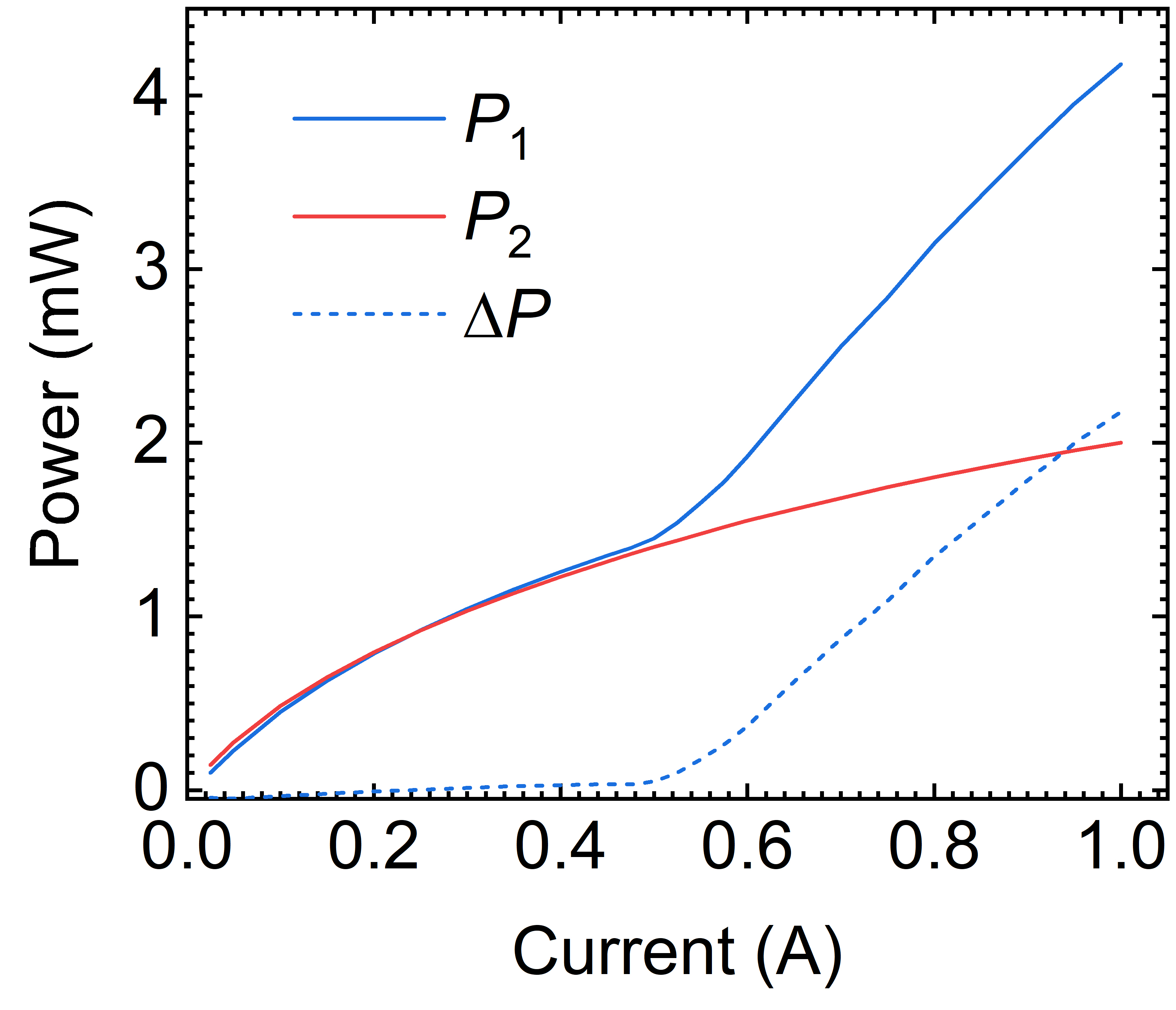


**Fig. S3 Light-current characteristics of the triple-lattice PCSEL under CW conditions.**

To avoid the spontaneous radiation interfering with the analysis of the lasing behavior, we both measure the light-current characteristics of constructively (corresponding to Fig. 6a) and destructively (corresponding to Fig. 6c) interacted triple-lattice PCSELs among the current range of 0-1 A, assigned as *P*_1_ and *P*_2_, respectively. *P*_2_ is used to evaluate the magnitude of spontaneous radiation because the injection current is far below its threshold current. ∆*P*, defined as the difference between *P*_1_ and *P*_2_, describes the lasing behavior of the constructively interacted triple-lattice PCSEL, as shown in Figure 4a.

It should be noted that the PCSELs were measured at 10 °C under CW conditions. This is because the PCSELs working at 1.55 μm have severe material absorption and are more sensitive to temperature.

To enable the device to operate at higher temperatures, we need to further reduce the device threshold current. Two approaches could be used. First, we need to improve the matching between the gain spectrum and the resonant peak. Second, we can optimize the filling factor of the photonic crystal, and adopt material with higher contrast to further reduce the optical loss of the resonator.


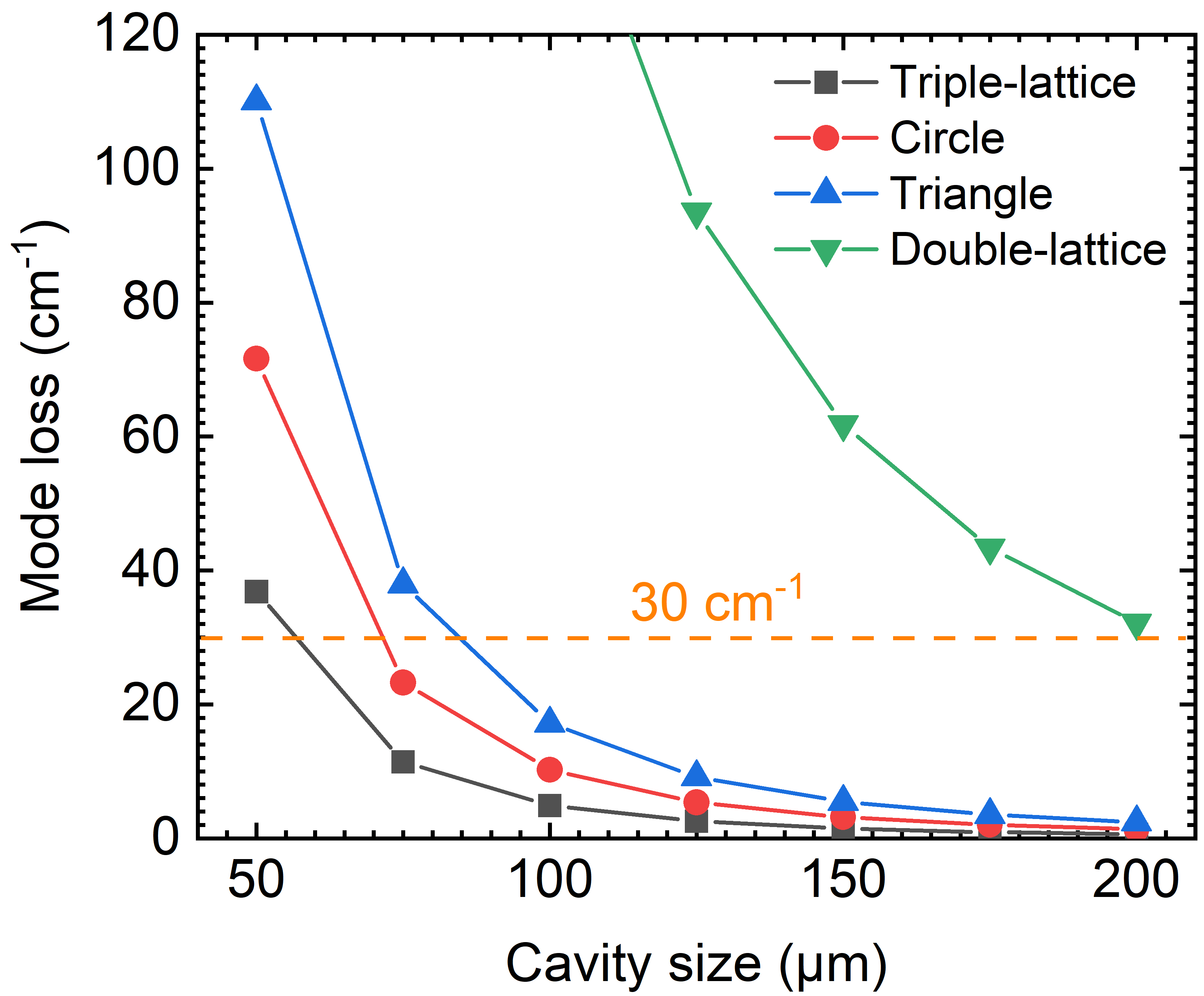


**Fig. S4 Mode loss as a function of cavity size** **for void-containing PCSELs**

As shown in Fig. S4, optical loss of the lowest-threshold mode increases rapidly with the decreasing cavity size. Among these resonators, the triple-lattice photonic-crystal resonator has the lowest optical loss, which gives us an approach to reduce the cavity size. Practically, the optical loss of the resonator needs to be controlled below 30 cm^-1^. A cavity smaller than 60 μm could be realized using the triple-lattice photonic crystal resonator. To obtain the same optical loss, the cavity size of the double-lattice PCSEL needs to be above 200 μm.

The all-semiconductor resonators in this work obey a similar regularity with their void-containing counterparts. However, the latter will have a lower optical loss than the former when they have the same cavity size due to their higher dielectric contrast. Thus, adopting the void-containing PCSEL would enable us to get a smaller laser if the manufacturability and reliability are solved.

**Table S2 Performance comparison of 1.55 μm PCSELs between our work and those in the literature.**

| **Year** | **Reference** | **Wavelength (μm)** | **Threshold** | **Maximum output power (mW)** | **Slope efficiency**  **(W A^-1^)** | **Structure** |
| --- | --- | --- | --- | --- | --- | --- |
| 2023 | this work | 1.55 | 1.66 kA cm^-2^ | 89 | 0.02 | all-semiconductor |
| 2023 | ref.5^5^ | 1.55 | 2.0 kA cm^-2^ | 120 | 0.056 | void-containing |
| 2022 | ref.6^6^ | 1.56 | 180 mA | 12.58 | 0.016 | FP coupled |
| 2020 | ref.7^7^ | 1.52 | 1.6 kA cm^-2^ | 0.5 | 0.002 | void-containing |

Table S2 lists the performance of 1.55 μm PCSELs reported in recent years. In our work, we adopt an all-semiconductor photonic-crystal resonator, which has a similar regrowth process as distributed feedback lasers (DFB). This makes the fabrication more manufacturable and reliable. However, our device’s threshold and output power are close to the air-contained PCSEL in ref. 5, and even much better than the result in ref.7. This demonstrates the validation of our design of the triple-lattice photonic-crystal resonator. The photonic-crystal resonator in ref.6 was fabricated from the surface of the epitaxial structure, and an F-P cavity with high-reflection coatings on both edge sides was required to enhance the optical feedback of the resonant mode.


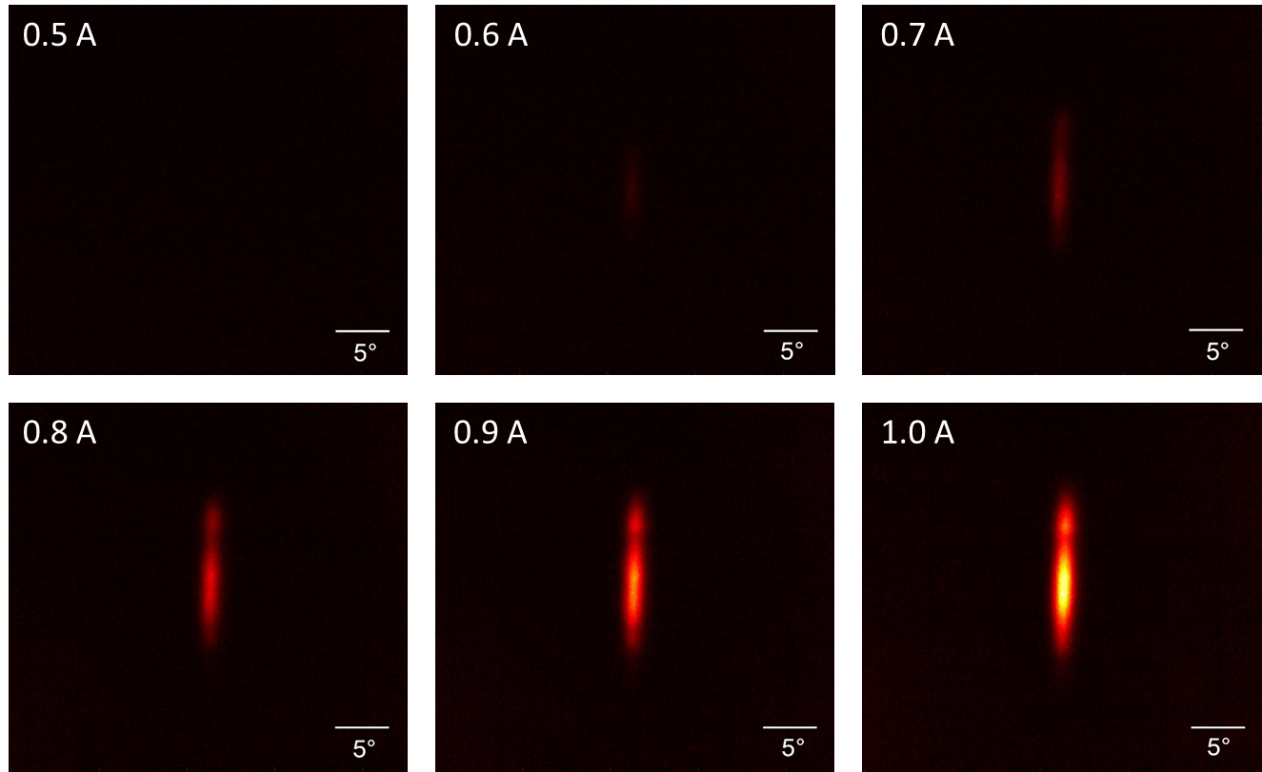


**Fig. S5 Far-field patterns at various injection currents under CW condition.**


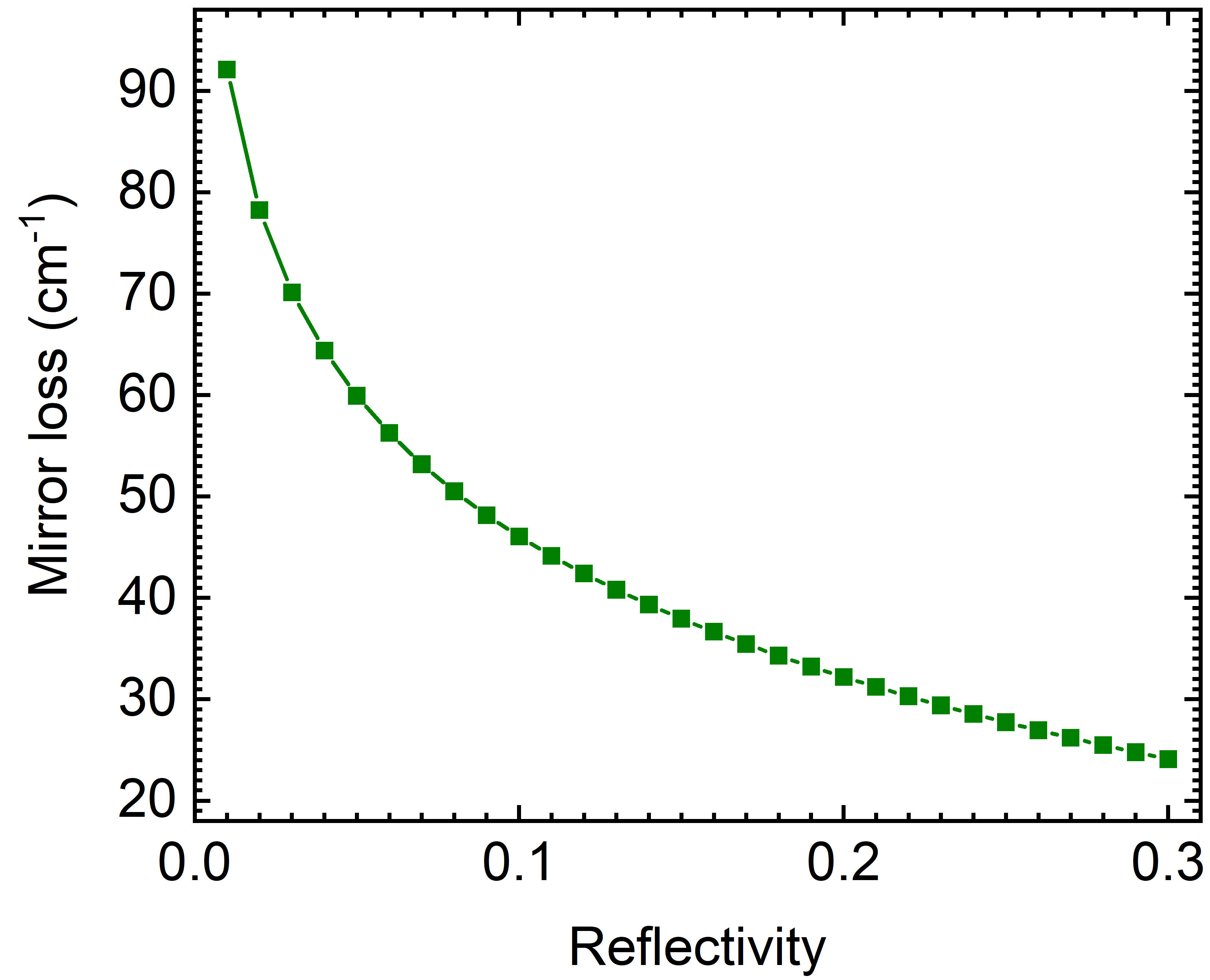


**Fig. S6 Mirror loss as a function of reflectivity.**

Figure S6 shows the mirror loss (or quality factor) of the FP modes with a cavity length of 500 μm. In our design, the optical loss of the lowest-threshold mode is about 28 cm^-1^. Thus, the potential mirror loss will be at the same magnitude as the modes intrinsic to the photonic-crystal resonator, if the reflectivity from the device edge is above 20%. The laser actually works in multi-mode.

To improve the far-field pattern, we need to reduce the number of lasing modes. For this goal, the optical feedback from the cleaved facet needs to be further suppressed, for instance, by introducing a deeper wet-etching mesa.

Besides, the triple-lattice resonator used in this paper reduces the threshold margin between different modes, while the optical losses of these modes are lowered. This is unfavorable for getting a single-mode laser. We also need to increase the threshold difference between fundamental modes and other modes in future, by adjusting the loss more accurately.


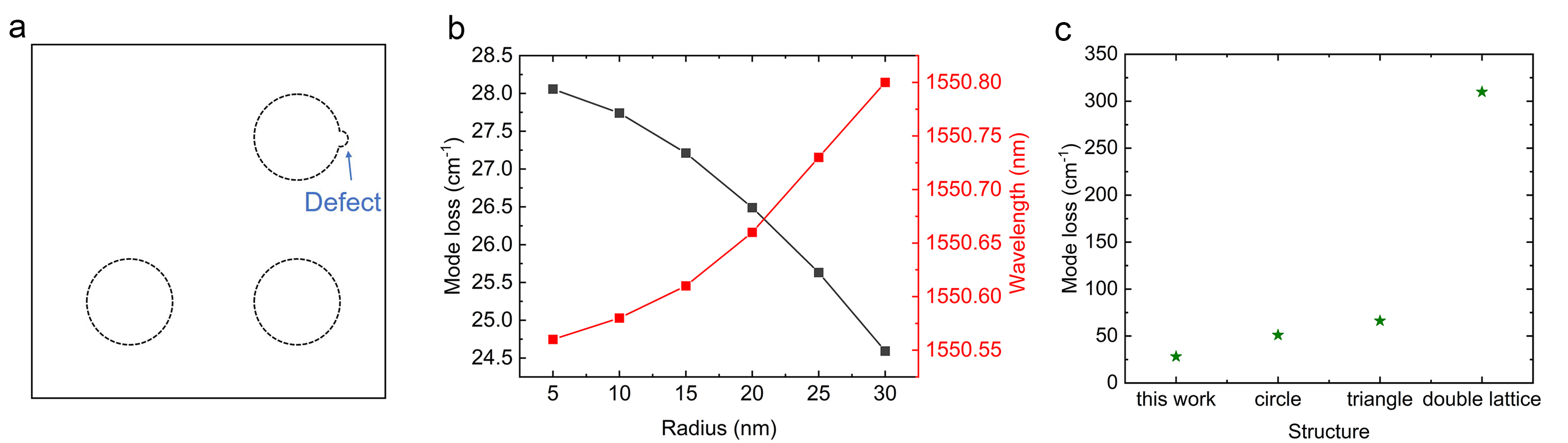


**Fig. S7 Evaluation of the influence of fabrication defect. a** Schematic illustrating the unit cell of photonic crystal with defect used in the calculation. **b** Mode loss and resonant wavelength as a function of the radius of the defect. **c** Mode loss of various photonic-crystal resonators with the same filling factor.

To quantitatively evaluate the effect of fabrication imperfection, we introduce a convex defect to the edge of the circular hole in our calculation, as shown in Fig. S7a. We study the influence of the defect on optical loss and resonant wavelength with increasing radius. The result is shown in Fig. S7b. As the defect radius increases from 5 nm to 30 nm, the optical loss of the lowest-threshold mode decreases by about 3.5 cm^-1^. It might be caused by the change of the filling factor. This magnitude could be neglected in contrast to the changes caused by the various types of resonators. The latter tends to change optical loss by tens, even hundreds per centimeter, as shown in Fig. S7c. However, considering the threshold margin (the mode loss difference between fundamental mode and other modes) is less than 0.1 cm^-1^ in our triple-lattice resonator, these imperfections will unavoidably have side effects on the stable operation of the lasing mode.

On the other hand, the imperfection on the sidewall might introduce some defects to the photonic crystal layer. Because this layer is adjacent to the active region, the defects could extend to the active layer through climbing and sliding, especially when working at high temperatures. This will lead to the degrading of the laser's quantum efficiency and output power.


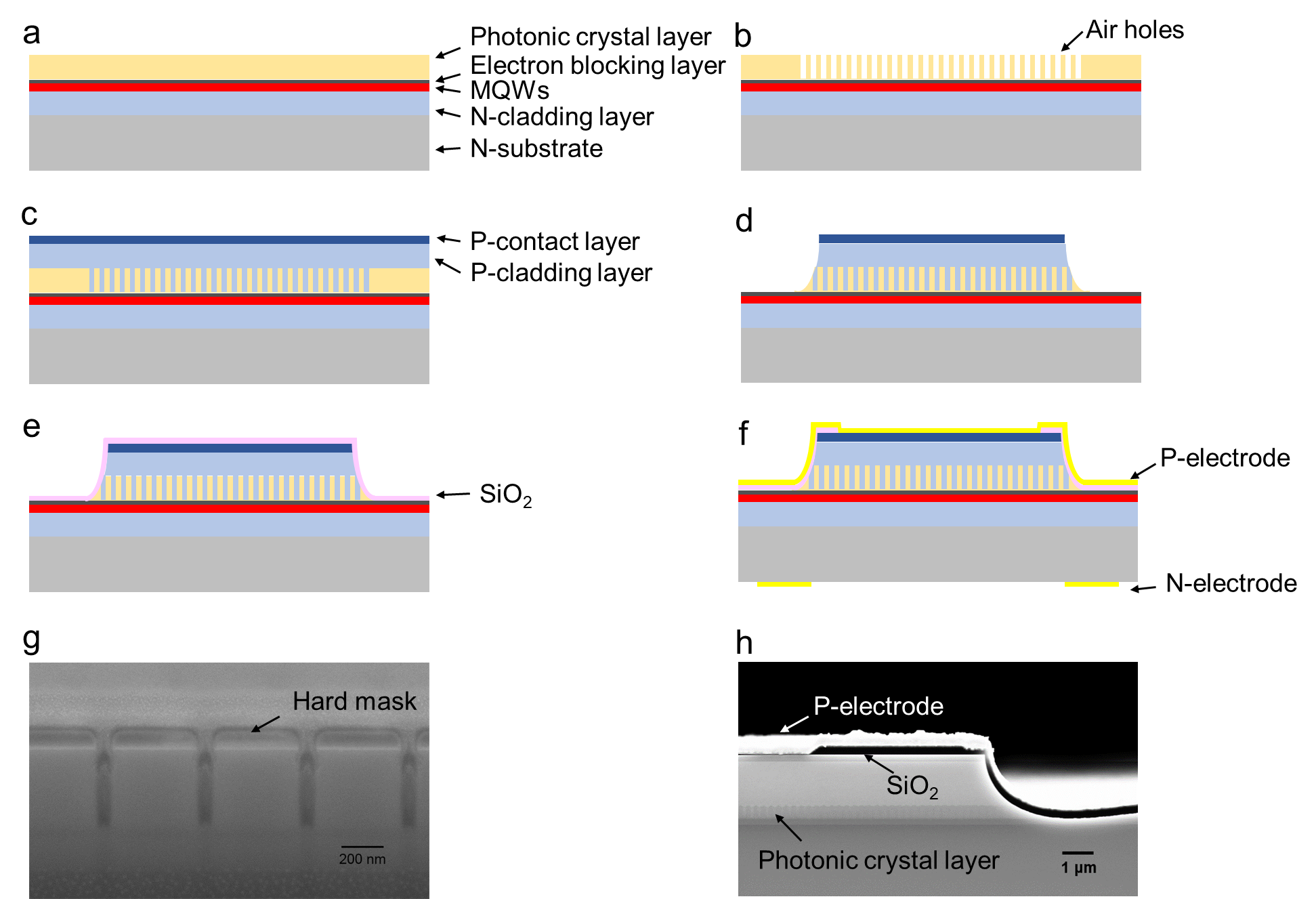


**Fig. S8** **Fabrication process of triple-lattice PCSEL.** **a** Growth of the epitaxial structure. **b** Fabrication of photonic-crystal resonator. **c** Regrowth on the pre-fabricated substrate. **d** Fabrication of p-mesa by wet etching. **e** Deposition of SiO_2_ electrical insulating layer. **f** Deposition of p-electrode and n-electrode. **g** Cross-sectional scanning electron microscope (SEM) image of the photonic-crystal resonator before regrowth. **h** SEM image of the edge of p-mesa.

**References**

1. Taylor, R. J. E. et al*.* All-Semiconductor Photonic Crystal Surface-Emitting Lasers Based on Epitaxial Regrowth. *IEEE Journal of Selected Topics in Quantum Electronics* **19**, 4900407-4900407 (2013).

2. Chua, S. L. et al. Larger-area single-mode photonic crystal surface-emitting lasers enabled by an accidental Dirac point. *Optics Letters* **39**, 2072-2075 (2014).

3. Yoshida, M. et al*.* High-brightness scalable continuous-wave single-mode photonic-crystal laser. *Nature* **618**, 727-732 (2023).

4. Sakai, K., Miyai, E. & Noda, S. Coupled-Wave Theory for Square-Lattice Photonic Crystal Lasers With TE Polarization. *IEEE Journal of Quantum Electronics* **46**, 788-795 (2010).

5. Hitaka, M. et al. 1.5 µm wavelength NPN-type photonic-crystal surface-emitting laser exceeding 100 mW. *Optics Express* **31**, 18645 (2023).

6. Wang, M. J. et al. Active beam steering enabled by photonic crystal surface emitting laser. Print at https://doi.org/10.48550/arXiv.2210.03500 (2022).

7. Bian, Z. J. et al*.* 1.5 μm Epitaxially Regrown Photonic Crystal Surface Emitting Laser Diode. *IEEE Photonics Technology Letters* **32**, 1531-1534 (2020).
